# RNAcentral 2021: secondary structure integration, improved sequence search and new member databases

**DOI:** 10.1093/nar/gkaa921

**Published:** 2020-10-27

**Authors:** RNAcentral Consortium, Blake A Sweeney, Anton I Petrov, Carlos E Ribas, Robert D Finn, Alex Bateman, Maciej Szymanski, Wojciech M Karlowski, Stefan E Seemann, Jan Gorodkin, Jamie J Cannone, Robin R Gutell, Simon Kay, Steven Marygold, Gil dos Santos, Adam Frankish, Jonathan M Mudge, Ruth Barshir, Simon Fishilevich, Patricia P Chan, Todd M Lowe, Ruth Seal, Elspeth Bruford, Simona Panni, Pablo Porras, Dimitra Karagkouni, Artemis G Hatzigeorgiou, Lina Ma, Zhang Zhang, Pieter-Jan Volders, Pieter Mestdagh, Sam Griffiths-Jones, Bastian Fromm, Kevin J Peterson, Ioanna Kalvari, Eric P Nawrocki, Anton S Petrov, Shuai Weng, Philia Bouchard-Bourelle, Michelle Scott, Lauren M Lui, David Hoksza, Ruth C Lovering, Barbara Kramarz, Prita Mani, Sridhar Ramachandran, Zasha Weinberg

**Affiliations:** European Molecular Biology Laboratory, European Bioinformatics Institute, Wellcome Genome Campus, Hinxton, Cambridge CB10 1SD, UK; Center for non-coding RNA in Technology and Health, Department of Veterinary and Animal Sciences, University of Copenhagen, Copenhagen, DK-1871, Denmark; Department of Integrative Biology, University of Texas at Austin, Austin, Texas 78712, USA; Department of Physiology, Development and Neuroscience, University of Cambridge, Downing Street, Cambridge CB2 3DY, UK; Department of Molecular Genetics, Weizmann Institute of Science, Rehovot 7610001, Israel; Department of Biomolecular Engineering, University of California Santa Cruz, Santa Cruz, CA, 95064, USA; Department of Haematology, University of Cambridge School of Clinical Medicine, Cambridge, CB2 0AW, UK; Università della Calabria, Dipartimento di Biologia, Ecologia e Scienze della Terra, Via Pietro Bucci Cubo 6/C, Rende, CS, 87036, Italy; DIANA-LAB, Department of Computer Science and Biomedical Informatics, University of Thessaly, Greece & Hellenic Pasteur Institute, 351 31, Greece; China National Center for Bioinformation & National Genomics Data Center, Beijing Institute of Genomics, Chinese Academy of Sciences, 100101, China; Cancer Research Institute Ghent (CRIG), 9000 Ghent, Belgium; Department of Biomolecular Medicine, Faculty of Medicine and Health Sciences, Ghent University, 9000 Ghent, Belgium; Faculty of Biology, Medicine and Health, University of Manchester, Oxford Road, Manchester, M13 9PT, UK; Science for Life Laboratory, Department of Molecular Biosciences, The Wenner-Gren Institute, Stockholm University, Stockholm, S-10691, Sweden; Department of Biological Sciences, Dartmouth College, Hanover, NH, 03755, USA; National Center for Biotechnology Information, National Library of Medicine, National Institutes of Health, Bethesda, MD 20894 USA; Center for the Origins of Life, School of Chemistry and Biochemistry, Georgia Institute of Technology, Atlanta, GA 30032, USA; Department of Genetics, Stanford University, Palo Alto, CA 94303, USA; Department of Biochemistry and Functional Genomics, Université de Sherbrooke, Sherbrooke, QCJ1H 5N4, Canada; Environmental Genomics and Systems Biology Division, Lawrence Berkeley National Laboratory, Berkeley, CA, 94720, USA; Department of Software Engineering, Faculty of Mathematics and Physics, Charles Univesity, Prague, 11800 Praha 1, Czech Republic; Functional Gene Annotation, Preclinical and Fundamental Science, UCL Institute of Cardiovascular Science, University College London, London, WC1E 6BT, UK; The Institute of Neuroscience, University of Oregon, Eugene, OR 97403-1254, USA; Bioinformatics Group, Department of Computer Science and Interdisciplinary Centre for Bioinformatics, Leipzig University, Härtelstraße 16–18, 04107, Leipzig, Germany; Department of Computational Biology, Adam Mickiewicz University in Poznan, Poznan, 61-614, Poland; The Biological Laboratories, Harvard University, 16 Divinity Avenue, Cambridge, MA 02138, USA

## Abstract

RNAcentral is a comprehensive database of non-coding RNA (ncRNA) sequences that provides a single access point to 44 RNA resources and >18 million ncRNA sequences from a wide range of organisms and RNA types. RNAcentral now also includes secondary (2D) structure information for >13 million sequences, making RNAcentral the world’s largest RNA 2D structure database. The 2D diagrams are displayed using R2DT, a new 2D structure visualization method that uses consistent, reproducible and recognizable layouts for related RNAs. The sequence similarity search has been updated with a faster interface featuring facets for filtering search results by RNA type, organism, source database or any keyword. This sequence search tool is available as a reusable web component, and has been integrated into several RNAcentral member databases, including Rfam, miRBase and snoDB. To allow for a more fine-grained assignment of RNA types and subtypes, all RNAcentral sequences have been annotated with Sequence Ontology terms. The RNAcentral database continues to grow and provide a central data resource for the RNA community. RNAcentral is freely available at https://rnacentral.org.

## INTRODUCTION

RNAcentral is the non-coding RNA (ncRNA) sequence database that currently integrates 44 specialist ncRNA databases, known as Expert Databases, to provide unified access to >18 million ncRNA sequences spanning a broad range of functions and species (1). In addition to sequences, RNAcentral provides a wide range of annotation types, such as genome coordinates, microRNA–target interactions (2,3), Gene Ontology (GO) terms (4), orthologs and paralogs (5), RNA family classification from Rfam (6) and more. Data can be accessed via text search, sequence similarity search, integrated genome browser and bulk data downloads from the FTP archive. The primary goal of RNAcentral is to provide open access to a comprehensive set of ncRNA sequences for a wide range of species, enabling the users to find what is known about individual sequences or download ncRNA sequences and their genomic locations that can be used for a broad range of studies, such as interpreting the results of RNA-seq experiments or training bioinformatic algorithms. RNAcentral also provides stable accessions for distinct RNA sequences, facilitating the work of other RNA resources.

**Figure 1.**
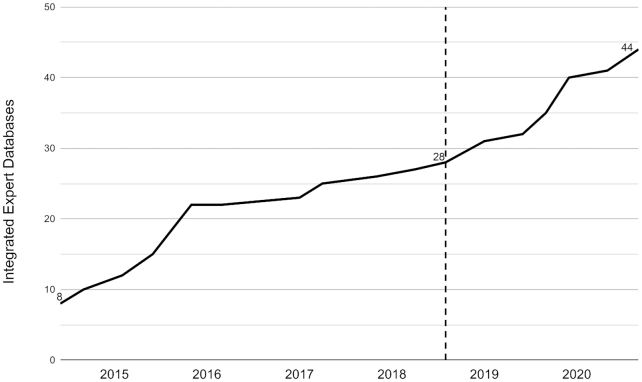
Growth in the number of RNAcentral Expert Databases since its launch in 2014 (for an up-to-date list see https://rnacentral.org/expert-databases). The previous NAR publication is marked with a vertical dashed line.

RNAcentral continues to grow (Figure 1) with the incorporation of 16 new Expert Databases since the last publication (1). In this paper, we discuss the new data and focus on the following major new features:

1. Newly integrated 2D structure information
2. Improved sequence similarity search
3. Transition to Sequence Ontology to annotate RNA types

## RNA 2D STRUCTURE INTEGRATION

Since 2017 RNAcentral has included 2D structure information starting with a tRNA dataset submitted by Genomic tRNA Database (GtRNAdb) (7). However, for the vast majority of RNAcentral sequences no secondary structure is available in the source database (e.g., ENA or RefSeq). In addition, there are accepted layouts and orientations for the display of secondary structures of well-known families (such as rRNA and tRNA) (8,9), but existing automated 2D visualization tools do not account for these layouts, making it difficult to analyze and compare structured RNAs. As these large families of well-known RNAs constitute the majority of sequences in RNAcentral, we set out to develop a new method for producing 2D structure diagrams in standard orientations called R2DT (RNA 2D Templates) (10).

The R2DT software automatically selects the best matching template from a library of 3632 2D templates that represent a wide range of RNA types, such as rRNA (both small and large subunit), tRNA, as well as 2675 RNA families from Rfam. A template encapsulates a reference sequence along with cartesian coordinates for each nucleotide and a 2D structure. The best-matching templates are selected using the Ribovore (https://github.com/nawrockie/ribovore) and tRNAscan-SE 2.0 (11) software, and are visualized using Traveler (12). The templates ensure that similar sequences are visualized in consistent, reproducible orientations and can be easily compared across related RNAs.

**Figure 2.**
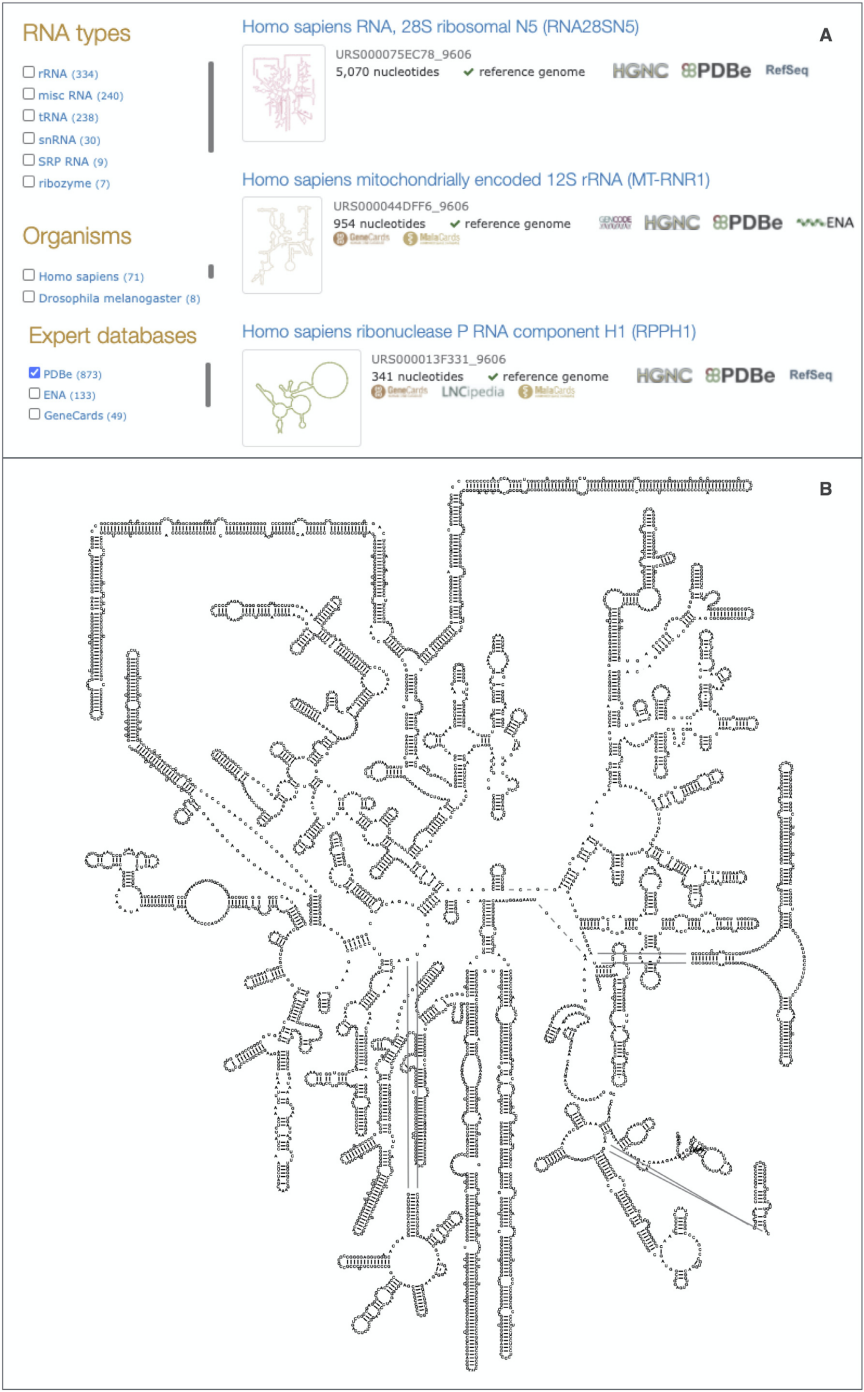
(**A**) The RNAcentral text search results include simplified thumbnails representing the 2D structures. (**B**) A 2D structure of the human LSU rRNA displayed on the sequence report page for URS000075EC78_9606.

A key strength of the method is the ability to visualize some of the largest structured RNAs, such as the human large subunit ribosomal rRNAs (LSU) with >5000 nucleotides (Figure 2). The LSU templates are displayed using a set of new 3D structure based templates from RiboVision (13). In addition, RiboVision provided a set of 3D structure based small subunit (SSU) rRNA templates that improves the representation of species-specific expansion segments in rRNA.

R2DT is now routinely applied to all sequences in RNAcentral. In the most recent release (version 16), we generated >13 million 2D structure diagrams, representing the world’s largest collection of RNA 2D structures. The 2D structures are displayed in the sequence report pages and in the text search results (Figure 2). In addition, R2DT is available as a web server (https://rnacentral.org/r2dt) that enables users to submit sequences and generate 2D diagrams.

As new templates are added to the R2DT library (e.g., with future Rfam releases), the number and quality of the 2D diagrams will be improved in RNAcentral. We welcome feedback about individual 2D structures to help prioritize improvements in R2DT.

## UPDATED SEQUENCE SIMILARITY SEARCH

Since 2015, RNAcentral has been hosting a sequence similarity search tool powered by the nhmmer software (14), to enable users to compare any query sequence against a comprehensive collection of ncRNAs (https://rnacentral.org/sequence-search). As RNAcentral grew in size, the search time increased and users experienced wait times of up to an hour to get the results. In 2019, an updated version of the search was launched using a scalable cloud infrastructure hosted at the Embassy Cloud platform provided by EMBL-EBI. The searches are executed in parallel and complete more quickly. For example, we repeated all searches submitted in 2019 using the new infrastructure and saw a decrease in the average search time from 4.5 min to 13 s, an approximately 20-fold increase in speed. Since the new launch was launched, the number of searches increased from around 600 to 3000 searches per month.

**Figure 3.**
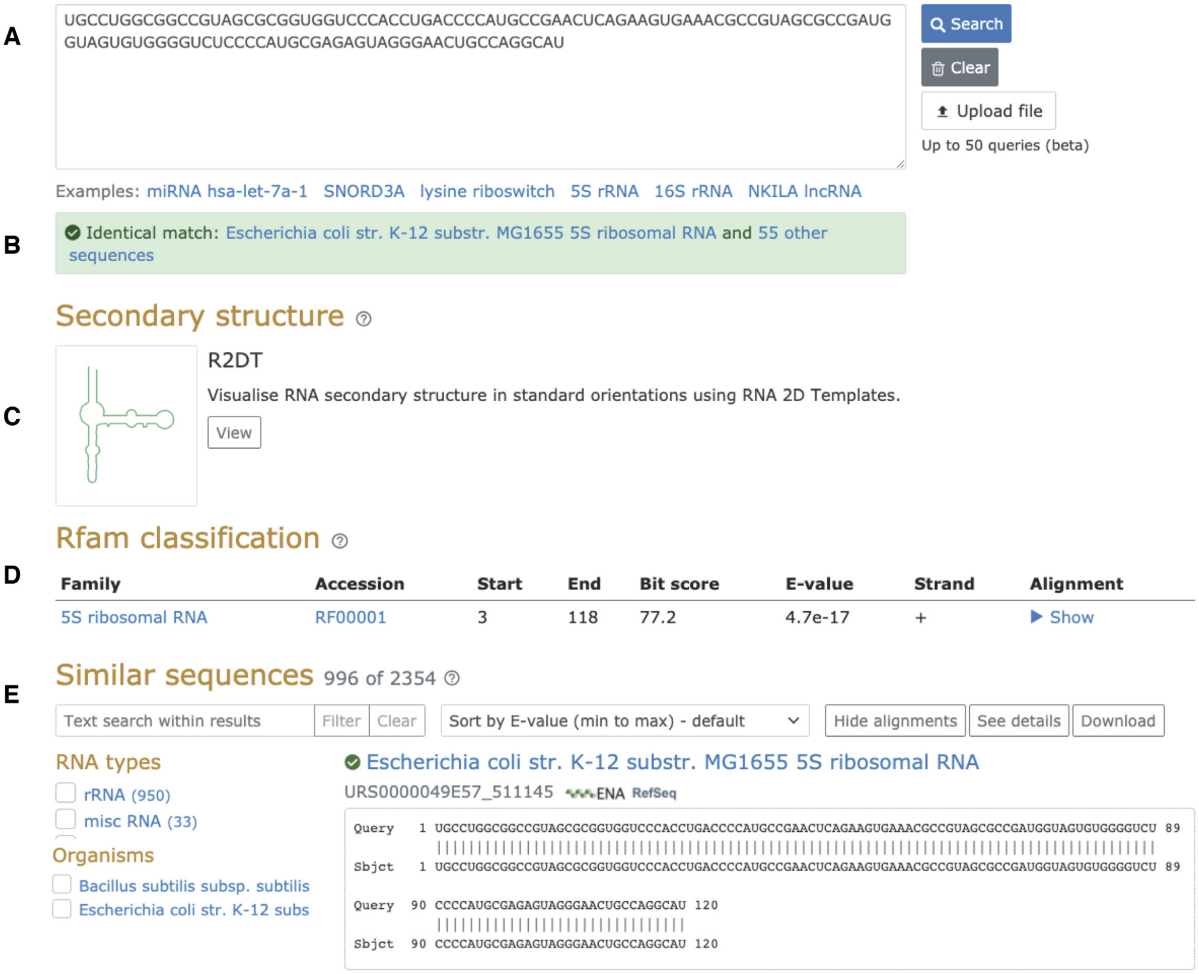
New RNAcentral sequence similarity search interface. (**A**) Query sequence, (**B**) Identical sequence found in RNAcentral, (**C**) Secondary structure visualized with R2DT, (**D**) Rfam classification, including Rfam family annotations and an alignment between the query and the Rfam model, (**E**) Similar sequences found in RNAcentral that can be filtered using facets, such as RNA types and Organisms, sorted or downloaded.

The new search features an updated interface that enables exploring the results using facets, such as species, RNA type and source database (Figure 3). The results can also be filtered by any keyword, similar to the RNAcentral text search, and sorted by *E*-value, sequence identity, query and target coverage and other parameters.

The query sequence is also automatically searched against the Rfam families (3024 as of Rfam 14.2) using Infernal (15). The Rfam results are post-processed to select the top scoring families from the same Rfam clan (16). For example, a rRNA sequence may match both eukaryotic and bacterial Rfam families, but the clan competition procedure keeps only the top scoring family. In addition, the sequence search is integrated with the R2DT software described above so that a 2D structure (if available) is visualized alongside similar sequences (Figure 3C). The updated search includes some of the most frequently requested features that were not available previously. For example, a batch search mode enables users to submit a FASTA file with up to 50 sequences in order to launch multiple searches simultaneously. Users can also download the results in several formats, including plain text and JSON.

The new interface is implemented as a reusable web component, enabling other RNAcentral Expert Databases or anyone else to include it in their websites to provide sequence similarity search to their users. The embeddable component is available at https://github.com/rnacentral/rnacentral-sequence-search-embed. It can be integrated into any website with a few lines of code. The component is highly customizable, for example, it is possible to select a subset of RNAcentral sequences to be searched or adjust the widget appearance to match the host website.

The search has been integrated into Rfam (6), miRBase (17) and snoDB (18). For example, when a user enters a query sequence in Rfam, it is not only annotated with Rfam families but also searched against a comprehensive set of sequences from RNAcentral. If a query comes from an RNA sequence not represented in Rfam, the results will include hits from RNAcentral, and if a query matches Rfam, the users will get additional information about matching sequences and can explore them using the facets.

In addition, in response to the COVID-19 pandemic, the cloud-centric approach enabled us to rapidly repurpose the RNAcentral infrastructure to search *Betacoronavirus* genomes instead of ncRNA sequences. The *Betacoronavirus* search provides virus-specific facets that enable filtering the results by virus, such as SARS-CoV or SARS-CoV-2, as well as the country of sample origin. The *Betacoronavirus* sequence search is available at https://covid19sequencesearch.github.io.

## REFINED RNA TYPE ASSIGNMENT USING SEQUENCE ONTOLOGY

Since its inception, RNAcentral has used the INSDC feature table (http://www.insdc.org/files/feature_table.html) and ncRNA vocabulary (http://www.insdc.org/rna_vocab.html) to annotate sequences with different RNA types. However, the INSDC classification lacks precision and does not distinguish between different rRNA types, such as SSU, LSU or 5S rRNAs, simply grouping them in a single category. Similarly, there were no specific terms for precursor microRNAs to separate them from other RNA precursors and maintain their connection with mature microRNAs. However, the Sequence Ontology (SO) (19) provides more granular terms for rRNAs, microRNAs and other RNA types.

**Figure 4.**
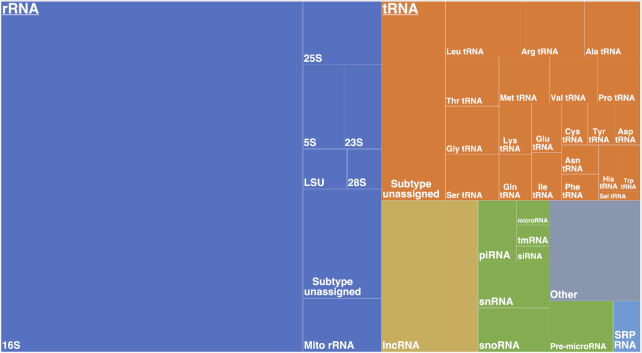
The distribution of RNAcentral sequences by the SO terms.

Several member databases, such as FlyBase or miRBase, already provide SO terms for their sequences. However, <10% of sequences have been annotated with SO terms at the time of submission to RNAcentral. We implemented a classification system that combines the information about the INSDC RNA types submitted by member databases, Rfam annotations and other information to expand the SO term coverage to the entire set of sequences found in RNAcentral. For example, for rRNA sequences, the R2DT rRNA template matches are used to transfer the corresponding SO term to the sequence, enabling the classification of rRNA subclasses. Consequently, an *Arabidopsis thaliana* sequence URS0000AF5D55_3702 previously annotated as misc_RNA in ENA is now assigned the SO term for 25S LSU rRNA due to matches to the eukaryotic large subunit (LSU) rRNA Rfam model (RF02543) and an eukaryotic LSU R2DT template. For the ‘other’ and ‘misc_RNA’ INSDC sequence classes, we use Rfam family annotations to assign the corresponding SO term to the sequences. For all remaining sequences, we map the INSDC RNA types to the SO terms using the mapping developed by the SO and the RefSeq groups (https://github.com/The-Sequence-Ontology/SO-Ontologies/issues/378). The resulting distribution of RNAcentral sequences by SO terms is shown in Figure 4.

## NEW DATA AND ANNOTATIONS

Since the last publication, the number of imported databases increased from 28 to 44 databases, integrating 16 additional resources listed in Table 1.

**Table 1.** Sixteen new member databases incorporated into RNAcentral in releases 11–16

Database	Description	Number of annotated sequences
5SrRNAdb	5SrRNAdb is an information resource for 5S ribosomal RNAs.	11 415
CRS	Conserved RNA structures (CRS) are structured elements of RNA molecules that are conserved across vertebrate species.	250 867
CRW	CRW Site provides comparative sequence and structure information for ribosomal, intron and other RNAs.	948
Ensembl Fungi, Metazoa, Protists	The Ensembl Genomes divisions complement the Ensembl database.	16 331; 64 237; 5652
GeneCards	GeneCards is a searchable, integrative database that provides comprehensive, user-friendly information on all annotated and predicted human genes.	250 702
IntAct	IntAct provides a freely available, open source database system and analysis tools for molecular interaction data. All interactions are derived from literature curation or direct user submissions.	382
LncBase	LncBase is a database of experimentally verified and computationally predicted microRNA targets on lncRNAs.	1337
LncBook	LncBook is a curated knowledgebase of human lncRNAs.	268 848
MalaCards	MalaCards integrates manually curated and text-mining sources to associate genes, including ncRNAs, with diseases, and lists the supporting evidence.	34 087
MirGeneDB	MirGeneDB is a curated microRNA gene database covering 45 metazoan organisms.	29 681
snoDB	snoDB is an interactive database of human snoRNA sequences, abundance and interactions.	1984
snoRNA Database	The snoRNA Database is a curated collection of archaeal snoRNAs maintained by the Lowe Lab at UC Santa Cruz.	727
ZFIN	The Zebrafish Information Network (ZFIN) is the database of genetic and genomic data for the zebrafish as a model organism.	1060
ZWD	ZWD is a collection of non-coding RNA alignments maintained by Zasha Weinberg.	47 998
		**Total: 986 256**

To provide detailed human ncRNA annotations, we imported data from **LncBook** (20) and **snoDB** (18) that host a variety of annotations for lncRNAs and snoRNAs, respectively. **GeneCards** (21) and **MalaCards** (22) have also been included into RNAcentral. GeneCards is a human gene knowledgebase, which aims to consolidate information about all human genes, coding and non-coding. MalaCards is an integrated database of human diseases and their annotations. MalaCards uses text mining and manual curation to associate human ncRNAs with information about diseases and lists the supporting literature. Notably, snoDB and GeneCards are also using RNAcentral as a data source. GeneCards also used RNAcentral to produce a comprehensive and non-redundant gene-centric view of ncRNAs, which is available at the ‘GeneCards ncRNAs’ track hub at the UCSC genome browser (23).

We completed the integration of all model organism databases forming the Alliance of Genome Resources (24) by importing **ZFIN** (25), a model organism database that hosts a wide array of expertly curated, organized and cross-referenced research data for zebrafish (*Danio rerio*). In order to provide genomic annotations for a broad range of organisms, we also imported ncRNAs from **Ensembl Fungi, Metazoa and Protists** (26).

We have added several new sources of functional annotations. We have integrated **IntAct** (27) bringing in 1152 intermolecular interactions for 382 RNAs, with the majority of data points coming from human and yeast (168 and 114 annotated RNAs, respectively). As curators continue to annotate additional interactions in IntAct, the new data will automatically flow into RNAcentral. We have also integrated microRNA–lncRNA interactions from **LncBase** v2 (3).

In addition to the automatic GO annotations created by RNAcentral, over 3400 ncRNAs currently are associated with GO terms, following the manual curation of research articles by the GO Consortium (1). Over 80% of these, 17 000 annotations capture the cellular role of human and mouse ncRNAs; microRNAs are the most commonly curated ncRNA. The majority of these annotations describe ‘gene silencing by miRNA’ and ‘mRNA binding’ and include the target of the ncRNA in the annotation extension field. However, downstream processes such as ‘regulation of epithelial-to-mesenchymal transition’ and ‘regulation of inflammatory response’ (28,29) are also described. All GO Consortium ncRNA annotations are available in RNAcentral, as well as via the GO browsers QuickGO and AmiGO and in other major resources including Ensembl, NCBI Gene, miRBase and the web service PSICQUIC.

Several RNA type specific databases have been included, such as **5SRNAdb** with 5S rRNAs (30), **snoRNA Database** with archaeal snoRNAs (31,32), **MirGeneDB** with mature and precursor microRNAs (33), as well as **CRW** with 5S, SSU and LSU rRNAs (8). A broad range of prokaryotic ncRNAs has been incorporated from the **ZWD** database (34), which includes high-quality sequence alignments for structured RNAs discovered in a diverse range of habitats and organisms.

We have also imported the Conserved RNA Structure (**CRS**) resource that computationally screened the human centered 100-way vertebrate sequence alignment from UCSC Genome Browser for conserved RNA secondary structures with CMfinder (35). We have integrated CRSs with a false discovery rate lower or equal to 10% in 29 vertebrate species and excluded matches to known structured RNAs from Rfam.

### Significant data updates

A number of previously integrated resources have provided significant updates in the last 2 years. Recent changes in **SILVA** (36) allowed us to integrate the SILVA-based inferred bacterial taxonomy into RNAcentral, which is displayed on the sequence report pages.

**FlyBase** (37) ncRNA annotations have been continuously updated within RNAcentral. Notably, FlyBase now reflects gene model annotations for *Drosophila melanogaster* only, meaning that ncRNA data for non-melanogaster genomes are no longer submitted by FlyBase to RNAcentral. However, ncRNA annotations for other Drosophila species are still available in RNAcentral as they are imported directly from NCBI/RefSeq and the ENA.

The HUGO Gene Nomenclature Committee (**HGNC**) (38) is the only organization with the authority to approve human gene symbols, including for ncRNA genes. Since January 2019, the HGNC has primarily worked on expanding its lncRNA dataset and has approved 528 new gene symbols, representing an increase of 11% for these genes. Note that the HGNC only provides one name per lncRNA gene without naming separate non-coding isoforms. Where possible, lncRNA genes have been named based on functional data from publications. Recent examples include *CHASERR* (39), *MYOPARR* (40) and *CEROX1* (41,42). Where no published data are available, the HGNC prioritizes naming lncRNA genes that have been manually annotated by both the RefSeq and Ensembl-Havana projects. These lncRNA genes are named based on genomic context using a systematic schema, as outlined in (43). The HGNC has also increased its small nuclear RNA dataset by 13% and its transfer RNA dataset by 2.5%.

With the most recent release of lncRNA database **LNCipedia** (version 5.2), significant efforts have been made to expand the functional annotation of lncRNAs in the database (44). By combining manual and programmatical curation of thousands of lncRNA papers in PubMed, 2482 PubMed articles were associated with lncRNAs in LNCipedia. As a result, LNCipedia currently contains 1555 unique lncRNA genes with at least one published article. In addition, improvements have been made to uniquely link LNCipedia entries with those of other databases such as Ensembl (45) and HGNC (38).

## OTHER IMPROVEMENTS

The RNAcentral website has been continuously updated with new features, such as the inclusion of the information about paralogs and orthologs from the Ensembl Compara pipeline (5). To increase discoverability with search engines, automatically generated summaries have been added for all sequences. The RNAcentral users can also display the miRBase word clouds (17) based on literature mining, which allows the users to see related terms at a glance. For example, microRNA mir-100 (URS000054969A_9606) is associated with cancer, with this term prominently featured in the word cloud.

Following user requests, RNAcentral now hosts a public Postgres database that provides the same data as the RNAcentral website. The database is meant to help users who would like to access RNAcentral programmatically or are interested in tasks that are not yet supported by the website. The connection details, example queries and a sample Python script can be found in (46) and at https://rnacentral.org/help/public-database.

## CONCLUSIONS

The RNAcentral database continues to grow in size and increase its utility. The addition of the 2D structure for a wide range of RNAs fills an important gap, as the users are now able to access not only the primary sequences but also the base pairing information and the 2D structure visualizations. The improved sequence search is faster and more user-friendly, and the embeddable search component is available for use on any website, enabling an ecosystem of RNAcentral member databases to reuse the resources in a cost-efficient way. The SO integration enables more granular annotation of ncRNAs and powers new ways of discovering the data using text search. The development of the next versions of RNAcentral is underway, focusing on the gene-centric organization of ncRNA transcripts and automatic incorporation of the latest scientific literature using text mining. We aim to continue integrating additional member databases, with 12 databases pending import, and we invite the developers of RNA databases wishing to join the RNAcentral Consortium to get in touch at https://rnacentral.org/contact.

## DATA AVAILABILITY

All data are freely available at https://rnacentral.org. The data can be accessed in the FTP archive, as well as through an API and a public Postgres database (see https://rnacentral.org/help for instructions). The code is available at https://github.com/rnacentral under the Apache 2.0 license.
